# Severe bacterial infection in thalassemia patients: prevalence, predisposing factors, causative organisms and outcomes

**DOI:** 10.3389/fmed.2026.1755729

**Published:** 2026-02-18

**Authors:** Ruttanaporn Taya, Adisak Tantiworawit, Harit Thongwitokomarn, Sirichai Srichairatanakool, Teerachat Punnachet, Nonthakorn Hantrakun, Pokpong Piriyakhuntorn, Thanawat Rattanathammethee, Sasinee Hantrakool, Chatree Chai-Adisaksopha, Ekarat Rattarittamrong, Lalita Norasetthada, Piangrawee Niprapan, Kanda Fanhchaksai, Pimlak Charoenkwan

**Affiliations:** 1 Department of Internal Medicine, Faculty of Medicine, Chiang Mai University, Chiang Mai, Thailand; 2 Division of Hematology, Department of Internal Medicine, Faculty of Medicine, Chiang Mai University, Chiang Mai, Thailand; 3 Division of Infectious Disease, Department of Internal Medicine, Faculty of Medicine, Chiang Mai University, Chiang Mai, Thailand; 4 Division of Hematology and Oncology, Department of Pediatrics, Faculty of Medicine, Chiang Mai University, Chiang Mai, Thailand

**Keywords:** anemia, bacterial infections, *Escherichia coli*, *Klebsiella pneumoniae*, thalassemia

## Abstract

**Introduction:**

Thalassemia is one of the most common genetic blood disorders globally. Bacterial infections remain a major cause of death among affected patients. To determine prevalence, predisposing factors, causative organism, and outcomes of severe bacterial infection in thalassemia patients.

**Methods:**

This retrospective study analyzed data from the Thalassemia Registry of the Division of Hematology, Department of Internal Medicine, Faculty of Medicine, Chiang Mai University (September 2013–September 2023). Thalassemia patients aged >15 years were included. Risk factors for severe bacterial infection were identified using multivariate logistic regression. Severe bacterial infection was defined as community-acquired involving a major organ, requiring parenteral antibiotics and/or surgery, and associated with a National Early Warning Score (NEWS) > 4.

**Results:**

A total of 208 patients were enrolled (mean age 45.3 ± 16.0 years; 62.0% female; 56.7% transfusion-dependent; 36.1% splenectomy). Severe bacterial infection occurred in 43 patients (20.7%). Primary bacteremia was the most common (23.2%), with *Klebsiella pneumoniae* (20.9%) and *Escherichia coli* (13.9%) as the leading pathogens. Infection-related mortality rate was 9.3%. Significant risk factors included hematocrit <21% (OR = 3.15; 95% CI 1.32–7.50; *p* = 0.01), splenectomy >10 years (OR = 2.46; 95% CI 1.07–5.69; *p* = 0.035), diabetes mellitus (OR = 10.42; 95% CI 2.21–49.12; *p* = 0.03), and liver hemochromatosis (OR = 3.76; 95% CI 1.64–8.63; *p* = 0.002).

**Conclusion:**

Severe bacterial infections affected 20.7% of thalassemia patients in this cohort, mainly bacteremia due to *Klebsiella pneumoniae* and *Escherichia coli.* Major risk factors were severe anemia, prolonged splenectomy, diabetes mellitus, and iron overload with liver hemochromatosis.

## Introduction

Thalassemia is an inherited anemia that is highly prevalent in northern Thailand with alpha thalassemia being the most common subtype. Bacterial infection is a major cause of death among patients with thalassemia (1–3). Factors contributing to infection include iron overload, continuous alloantigen stimulation from multiple blood transfusions, zinc deficiency, and reduced immune clearance capacity following splenectomy (4–6).

Severe infections are a major complication in patients with thalassemia, resulting from multiple interacting risk factors (7, 8). Iron overload secondary to repeated blood transfusions impairs immune cell function and promotes bacterial proliferation (9). Splenomegaly and hypersplenism lead to increased destruction of white blood cells, while splenectomy removes a critical immune organ, predisposing patients to infections caused by encapsulated organisms (7, 10, 11). Chronic anemia and malnutrition further compromise host immunity (12). In addition, frequent hospital visits and transfusions increase exposure to healthcare-associated pathogens (13). Collectively, these factors render patients with thalassemia significantly more susceptible to serious bacterial infections than the general population (13).

Previous studies have reported bacterial infections in thalassemia patients caused predominantly by *Escherichia coli*, and *Klebsiella pneumoniae* (8, 14–16). *Burkholderia pseudomallei* has also been identified as a pathogen in the Northeastern region of Thailand (14). Septicemia is the most common severe infectious complication (8, 14, 15). Evaluated serum ferritin levels, a rapid decline in hemoglobin of more than 2 g/dL per week, diabetes mellitus, and a history of splenectomy have been identified as a risk factors associated with increased mortality (1, 10, 15).

Most previous studies have focused on individual thalassemia subtypes, with limited comprehensive analyses encompassing all variants. This gap is especially evident in alpha thalassemia patients, where data remain particularly limited. Moreover, the definitions of bacterial infection vary across studies, and the risk factors contributing to severe bacterial infections in thalassemia patients have not been fully elucidated. Therefore, this study aims to investigate both alpha and beta thalassemia patients, with a specific focus on severe bacterial infection.

## Materials and methods

### Ethical approval

The study was consisted with the 1975 Declaration of Helsinki on Ethical Principles for Medical Research Involving Human Subjects. The study was approved by the Institutional Research Ethics Committee at the Faculty of Medicine, Chiang Mai University (Study approved number: MED-2566-0202). Informed consent was waived as the study involved no more than minimal risk.

Data were retrospective reviewed from the Thalassemia Registry and medical records of the Division of Hematology, Faculty of Medicine, Chiang Mai University, covering the period from September 2013 to September 2023. Collected data included clinical characteristics, infection site, causative organisms, and clinical outcomes. Baseline laboratory parameters prior to infection were obtained, including completed blood count (CBC), serum ferritin, liver iron concentration (LIC), and cardiac MRI T2*.

Severe infection was defined as a community-acquired bacterial infection involving a major organ and requiring parenteral antibiotics administration and/or surgical intervention, with a National Early Warning Score (NEWS) greater than 4 (17). The diagnosis was based on the clinical presentation, physical examination findings, and laboratory investigations confirmed by pathogen isolation from blood, pus, stool, cerebrospinal fluid or other body fluids (including negative culture with clinical evidence), as well as imaging studies. Baseline laboratory values were calculated as the mean of five routine blood tests collected before the onset of infection.

For incidence analysis, severe bacterial infection was assessed on a per-patient basis. Only the first episode of severe infection in each patient was included, and recurrent episodes were not counted separately.

### Patients

Eligibility participants included patients who diagnosed with thalassemia both alpha and beta subtypes confirmed by high-performance liquid chromatography (HPLC) and molecular testing, aged over 15 years. Patients with other risk factors for bacterial infection, such as HIV infection, malignancy, other hemolytic diseases or those receiving immunosuppressive therapy, were excluded.

### Outcomes

The primary endpoints of the study were the prevalence, causative organisms, sources of infection, and risk factors associated with severe bacterial infections. The secondary endpoint was the clinical outcome of severe bacterial infections, categorized as a recovery without intensive care unit (ICU) admission, recovery with ICU admission, or death.

### Statistical analysis

According to a previous study (16), the incidence of severe bacterial infection among transfusion-dependent thalassemia patients was 22.47%. With an allocation ratio of 3.5, a 95% confidence intervals (CI), and a test power of 80%, the required sample size was calculated to be 230.

Continuous variables with normally distribution were presented as mean, and standard deviation (SD), while those with a non -normal distribution were presented as median and interquartile range (IQR). Categorical variables were reported as percentages. Univariable logistic regression was first used to identify potential risk factors for severe bacterial infections, followed by multivariable logistic regression. Risk factors were presented as adjusted odds ratios (OR) with 95% CI. The prevalence, causative organisms, and infection sites were also described. Statistical significance was defined as *p* < 0.05. All statistical analyses were performed using STATA software, version 10 (StataCorp, College Station, TX, USA).

## Results

A total of 247 patients with thalassemia were enrolled in the study. Thirty-nine patients were excluded due to HIV infection, malignancy, immunosuppressive therapy, or other hemolytic diseases. A total of 208 patients with thalassemia were included, comprising 144 patients with beta-thalassemia and 64 patients with alpha-thalassemia.

Severe bacterial infections were identified in 43 of the remaining 208 patients, resulting in a prevalence rate of 20.7%. The prevalence of severe bacterial infection was 22.2% (32/144) in patients with beta-thalassemia and 17.2% (11/64) in those with alpha-thalassemia. Although patients with beta-thalassemia had a slightly higher incidence of severe bacterial infection compared with those with alpha-thalassemia, the difference was not statistically significant (*p* value = 0.408). The clinical characteristics of the study population are summarized in Table 1. The mean age in both infected and non-infected groups was 45 years, with a higher proportion of female patients. Diabetes mellitus and pulmonary hypertension were more frequent complications observed in the infected group. Beta-thalassemia/HbE disease was the most common subtype among all participants and was also the most predominant subtype in the infected group. Baseline hemoglobin levels were slightly lower in the infected group compared to the non-infected group, with a similar trend observed for hematocrit levels. Conversely, serum ferritin levels were higher in the infected group. Deferiprone was the most frequently used iron chelating agent, while the combination of deferoxamine and deferiprone was the most common dual iron chelating regimen, accounting for 17.6% of patients. Liver hemochromatosis was the most frequent site of iron overload, followed by involvement of the endocrine system.

**Table 1:** Clinical characteristics of thalassemia patients with severe bacterial infection.

Characteristics	Total (*n* = 208)	No infection (*n* = 165)	Infection (*n* = 43)
Age at enrollment, years, mean ± SD	45.3 ± 16.0	45.6 ± 16.9	44.2 ± 12.2
Sex, *n* (%)			
Female	129 (62.0)	103 (62.4)	26 (60.5)
Underlying disease, *n* (%)			
Hypertension	15 (7.2)	14 (8.5)	1 (2.3)
Diabetes mellitus	23 (11.1)	16 (9.7)	7 (16.3)
Heart failure	3 (1.4)	2 (1.2)	1 (2.3)
Pulmonary hypertension	17 (8.2)	10 (6.1)	7 (16.3)
HBV/HCV infection	16 (7.7)	13 (7.9)	3 (7.0)
Liver cirrhosis	6 (2.9)	3 (1.8)	3 (7.0)
Chronic kidney disease	6 (2.9)	5 (3.0)	1 (2.3)
Osteoporosis	46 (22.1)	38 (23.0)	8 (18.6)
Thalassemia type, *n* (%)			
Alpha-thalassemia	**64 (30.8)**	**53 (32.1)**	**11 (25.6)**
HbH disease with variants^a^	52 (81.3)	44 (83.0)	8 (72.7)
HbH with CS with variants^b^	12 (18.8)	9 (17.0)	3 (27.3)
Beta-thalassemia	**144 (69.2)**	**112 (67.9)**	**32 (74.4)**
Beta thalassemia major	26 (18.1)	18 (16.1)	8 (25.0)
Beta thalassemia/HbE disease	113 (78.5)	90 (80.3)	22 (71.9)
Homozygous HbE	3 (2.1)	2 (1.8)	1 (3.1)
Other^c^	2 (1.3)	2 (1.8)	0 (0.00)
Baseline laboratory			
Hemoglobin (g/dL), mean ± SD	7.64 ± 1.48	7.71 ± 1.51	7.36 ± 1.33
Hematocrit (%), mean ± SD	24.72 ± 5.02	25.16 ± 5.01	23.04 ± 4.76
White blood cell (×10^9^/L), (IQR)	8.05 (6.12, 13.39)	7.80 (6.00, 12.63)	10.07 (7.05, 14.77)
Platelet (×10^9^/L), (IQR)	273.75 (179.25, 533.25)	260.50 (179.00, 532.00)	369.00 (183.50, 545.50)
Serum ferritin (μg/mL), (IQR)	1047.6 (698.8, 2038.7)	938.8 (681.0, 1649.4)	1660.8 (854.3, 3989.2)
Splenectomy, *n* (%)			
Yes	75 (36.1)	52 (31.5)	23 (53.5)
Years after splenectomy, mean ± SD	25.3 ± 10.1	25.0 ± 10.2	26.0 ± 10.1
Iron chelating agent, *n* (%)			
Deferoxamine	65 (31.3)	46 (27.9)	19 (44.2)
Deferiprone	132 (63.5)	104 (63.0)	28 (65.1)
Deferasirox	36 (17.3)	31 (18.8)	5 (11.6)
Combine deferoxamine	56 (26.9)	41 (24.8)	15 (34.9)
Secondary hemochromatosis, *n* (%)			
Cardiac hemochromatosis	3 (1.4)	2 (1.2)	1 (2.3)
Endocrine	54 (26.0)	37 (22.4)	17 (39.5)
Liver hemochromatosis (LIC ≥ 7 mg/g)	69 (33.2)	46 (27.9)	23 (53.5)
EMH	31 (14.9)	22 (13.3)	9 (20.9)
Cardiac T2* (ms), mean ± SD	39.7 ± 8.2	40.7 ± 7.2	37.4 ± 9.9

a AE Bart’s, EH Bart’s.

b AE Bart’s CS, Bart’s CS.

c Beta thal/Hb Tak, Hb Jax/CS.

SD, standard deviation; IQR, interquartile range; HBV, Hepatitis B Virus; HCV, Hepatitis C Virus; LIC, liver iron concentration; EMH, extramedullary hematopoiesis.

**Figure 1:**
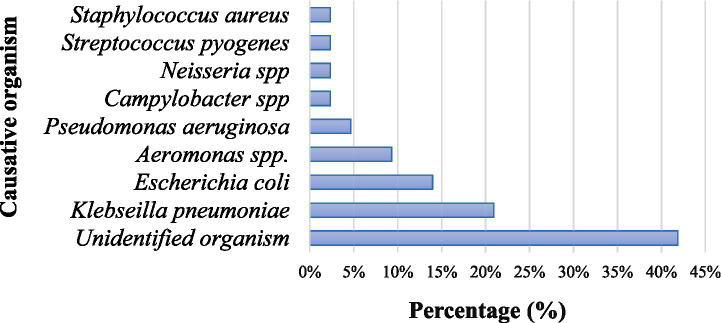
Causative organisms of severe bacterial infection.

Among the 43 patients with severe infections, unidentified organisms were the most common finding, reported in 18 cases (41.9%). The most frequently identified pathogen was *Klebsiella pneumoniae*, detected in 9 patients (20.9%), followed by *Escherichia coli* in 6 patients (14.0%). Less common pathogens included *Aeromonas species* in 4 cases (9.3%) and *Pseudomonas aeruginosa* in 2 cases (4.7%). Rare infections were caused by *Campylobacter species*, *Neisseria species*, *Streptococcus pyogenes*, and *Staphylococcus aureus*, with each identified in one patient (2.3%). Overall, Gram-negative bacteria were the predominant organisms of severe infections in this cohort. A considerable proportion of infections, particularly those involving the hepatobiliary tract infection (41.9%), had unidentified organisms (Figure 1).

The distribution of infection sites was as follows; bacteremia (23.2%), hepatobiliary tract (17.9%), genitourinary tract (16.1%), head and neck (12.5%), gastrointestinal tract (8.9%), sepsis of unknown origin (7.1%), respiratory tract (7.1%), skin and soft tissue (5.4%), and bone and joint (1.8%). The mortality rate attributed to bacterial infection was 9.3%.

Significant risk factors for severe bacterial infection are presented in Table 2. On multivariable logistic regression analysis, hematocrit less than 21%, splenectomy duration more than 10 years, diabetes mellitus, and liver hemochromatosis were identified as statistically significant predictors of severe infection. There was no significant association between pulmonary hypertension, transfusion-dependent thalassemia, deferoxamine used, and occurrence of severe bacterial infection.

**Table 2:** Univariable and multivariable logistic regression for factors associated with severe bacterial infection in patients with thalassemia.

Factors	Univariable logistic regression			Multivariable logistic regression		
	OR	95% CI	*p*-value	AOR	95%CI	*p*-value
Age at enrollment, years, mean ± SD	0.99	0.97–1.02	0.595			
Female	0.92	0.46–1.83	0.814			
Pulmonary hypertension	3.01	1.07–8.46	0.036	1.63	0.48–5.47	0.435
Transfusion-dependent thalassemia	2.02	0.98–4.15	0.05	0.82	0.31–2.19	0.694
Hematocrit ≤21%	2.56	1.23–5.32	0.012	3.15	1.32–7.50	**0.010**
Serum ferritin ≥1,000 μg/mL	2.95	1.42–6.15	0.004			
History of splenectomy	2.50	1.26–4.95	0.009			
Splenectomy >10 years	2.41	1.22–4.78	0.012	2.46	1.07–5.69	**0.035**
Deferoxamine	2.05	1.03–4.09	0.042	0.78	0.31–1.98	0.596
Diabetes mellitus	8.75	2.09–36.64	0.003	10.42	2.21–49.12	**0.003**
Liver hemochromatosis/LIC > 7	2.98	1.49–5.93	0.002	3.76	1.64–8.63	**0.002**

OR, odds ratio; AOR, adjusted odds ratio; CI, confidence intervals; LIC, liver iron concentration.

## Discussion

Bacterial infections have been frequently reported in patients with thalassemia, a condition characterized by several notable immune alterations. These include a reduction in neutrophil count, altered number and function of natural killer (NK) cells, increased CD8+ suppressor T cells activity, and impaired macrophage function. Additionally, abnormalities in chemotaxis and phagocytosis, as well as altered production of interferon-gamma (IFN-γ), have been documented, all of which contribute to the increased susceptibility to infections in these patients (10, 18).

The incidence of infection observed in this cohort was consistent with findings from previous study (16). This study included all types of thalassemia. Bacteremia was the most common type of infection, aligning with reports from Northeast Thailand and Taiwan (14, 16). *Escherichia coli* and *Klebsiella pneumoniae* remained the predominant pathogens responsible for bacterial infections in thalassemia patients. However, unlike prior study (14), no cases of *Burkholderia pseudomallei* infection were identified in our center, which may be explained by regional epidemiology, as this pathogen is uncommon in our region (19). Thalassemia patients exhibit a higher prevalence of gut-associated bacteria that are typically considered detrimental, leading to dysbiosis and increases the susceptibility to infection, particularly those caused by *Enterobacteriaceae* spp. (20). Reduced phagocytic activity due to iron overload and liver dysfunction may further predispose patients to *Klebsiella pneumoniae* infection (10, 21). No association between *Yersinia* infection and deferoxamine therapy was observed in our study, differing from previous reports (7, 22). This discrepancy may be explained by the limited use of deferoxamine in our cohort.

In contrast, head and neck infections were more prevalent in our study compared to previous research (13% vs. 5%) (23). The primary cause of deep neck infections was odontogenic, accounting for 40% of cases, with dental caries being the most common etiology, often related to poor oral hygiene (24). High iron deposition in the salivary glands leads to reduced phosphorus and IgA concentrations in saliva, as well as decreased saliva flow, further contributing to the risk of infection (25).

In our study, the significant predisposing factors of severe bacterial infection were lower pre-transfusion hematocrit, diabetes mellitus, splenectomy performed more than 10 years prior, and liver hemochromatosis. These findings are consistent with previous reports (1, 14).

Hemolysis increases levels of non-transferrin-bound iron, free heme, and heme oxygenase-1 (HO-1), which are associated with immune dysfunction and iron homeostasis dysregulation. Invasive bacteria also require iron for their metabolic and pathogenic processes (26, 27). We used liver hemochromatosis as a marker for iron overload rather than serum ferritin, as ferritin does not accurately reflect tissues iron accumulation. Excess iron not only promotes pathogen growth but also plays a critical role in modulating the immune response of the host. A history of splenectomy, especially more than 10 years earlier, is associated with a higher risk of infection with encapsulated bacteria due to impaired antibody response to new antigens, mediated by CD4^+^ T-cell dysfunction (10, 28, 29). Diabetes mellitus, a recognized complication of iron overload in thalassemia, was also identified as a significant risk factor for severe bacterial infection, consistent with prior findings (16). These risk factors may help to identify thalassemia patients at high risk of bacterial infections, allowing early detection and prompt antibiotic treatment to improve clinical outcomes.

The strength of our study lies in the inclusion of both alpha and beta thalassemia, enabling comprehensive analysis of the common type, causative organisms, and risk factors for severe bacterial infection. The proposed mechanisms and risk factors may help identify high-risk patients and guide strategies for infection prevention. However, several limitations should be noted. First, some data were missing for certain patients, including vaccination records. Second, other potential risk factors may not have been captured due to the retrospective nature of the study. Third, patient enrollment was limited by the number of eligible cases available during the study period, which may have slightly reduced the statistical power of the study. Therefore, further prospective studies with larger sample sizes are warranted to better explore those associations. Fourth, although serum ferritin levels were higher among patients with infections, C-reactive protein (CRP) and procalcitonin were not routinely measured in most patients in this cohort. Therefore, these biomarkers could not be analyzed in the present study. Additionally, variables such as time to antibiotic administration, appropriateness of antibiotic therapy, and hospital length of stay, should be included in further analyses to be understand their impact on outcomes.

## Conclusion

Severe bacterial infections occurred in 20.7% of thalassemia patients in this cohort, with *Klebsiella pneumoniae* and *Escherichia coli* being the predominant pathogens, most commonly presenting as bacteremia. The overall mortality rate was 9.3%. Major risk factors identified were severe anemia, prolonged splenectomy, diabetes mellitus, and liver hemochromatosis.

## Data Availability

The original contributions presented in the study are included in the article/supplementary material, further inquiries can be directed to the corresponding author.

## References

[1] Dhanya R Sedai A Ankita K Parmar L Agarwal RK Hegde S . Life expectancy and risk factors for early death in patients with severe thalassemia syndromes in South India. Blood Adv. (2020) 4:1448–57. doi: 10.1182/bloodadvances.2019000760 32282881 PMC7160270

[2] Tantiworawit A Kamolsripat T Piriyakhuntorn P Rattanathammethee T Hantrakool S Chai-Adisaksopha C . Survival and causes of death in patients with alpha and beta-thalassemia in northern Thailand. Ann Med. (2024) 56:2338246. doi: 10.1080/07853890.2024.2338246, 38604224 PMC11011226

[3] Teawtrakul N Jetsrisuparb A Pongudom S Sirijerachai C Chansung K Wanitpongpun C . Epidemiologic study of major complications in adolescent and adult patients with thalassemia in northeastern Thailand: the E-SAAN study phase I. Hematology. (2018) 23:55–60. doi: 10.1080/10245332.2017.1358845, 28759343

[4] Ganz T. Iron and infection. Int J Hematol. (2018) 107:7–15. doi: 10.1007/s12185-017-2366-2, 29147843

[5] Pieracci FM Barie PS. Iron and the risk of infection. Surg Infect. (2005) 6:S41–6. doi: 10.1089/sur.2005.6.s1-41 19284357

[6] Kao JK Wang SC Ho LW Huang SW Chang SH Yang RC . Chronic iron overload results in impaired bacterial killing of THP-1 derived macrophage through the inhibition of lysosomal acidification. PLoS One. (2016) 11:e0156713. doi: 10.1371/journal.pone.0156713, 27244448 PMC4886970

[7] Vento S Cainelli F Cesario F. Infections and thalassaemia. Lancet Infect Dis. (2006) 6:226–33. doi: 10.1016/S1473-3099(06)70437-6, 16554247

[8] Wanachiwanawin W. Infections in E-beta thalassemia. J Pediatr Hematol Oncol. (2000) 22:581–7. doi: 10.1097/00043426-200011000-00027, 11132234

[9] Chan GC Chan S Ho PL Ha SY. Effects of chelators (deferoxamine, deferiprone and deferasirox) on the growth of *Klebsiella pneumoniae* and *Aeromonas hydrophila* isolated from transfusion-dependent thalassemia patients. Hemoglobin. (2009) 33:352–60. doi: 10.3109/03630260903211888 19814682

[10] Taher AT Farmakis D Porter JB Cappellini MD Musallam KM. Guidelines for the Management of Transfusion Dependent β-Thalassaemia (TDT). 5th ed. Nicosia, (CY): Thalassaemia International Federation (2025). 40367250

[11] Ricerca BM Di Girolamo A Rund D. Infections in thalassemia and hemoglobinopathies: focus on therapy-related complications. Mediterr J Hematol Infect Dis. (2009) 1:e2009028. doi: 10.4084/MJHID.2009.028, 21415996 PMC3033166

[12] Gluba-Brzozka A Franczyk B Rysz-Gorzynska M Rokicki R Koziarska-Rosciszewska M Rysz J. Pathomechanisms of immunological disturbances in beta-thalassemia. Int J Mol Sci. (2021) 22:9677. doi: 10.3390/ijms22189677 34575839 PMC8469188

[13] Haque M Sartelli M McKimm J Abu Bakar M. Health care-associated infections—an overview. Infect Drug Resist. (2018) 11:2321–33. doi: 10.2147/IDR.S177247, 30532565 PMC6245375

[14] Teawtrakul N Jetsrisuparb A Sirijerachai C Chansung K Wanitpongpun C. Severe bacterial infections in patients with non-transfusion-dependent thalassemia: prevalence and clinical risk factors. Int J Infect Dis. (2015) 39:53–6. doi: 10.1016/j.ijid.2015.09.001, 26358855

[15] Peng CT Tsai CH Wang JH Chiu CF Chow KC. Bacterial infection in patients with transfusion-dependent beta-thalassemia in Central Taiwan. Acta Paediatr Taiwan. (2000) 41:318–21. 11198938

[16] Wang SC Lin KH Chern JP Lu MY Jou ST Lin DT . Severe bacterial infection in transfusion-dependent patients with thalassemia major. Clin Infect Dis. (2003) 37:984–8. doi: 10.1086/378062, 13130412

[17] Williams B. The National Early Warning Score: from concept to NHS implementation. Clin Med (Lond). (2022) 22:499–505. doi: 10.7861/clinmed.2022-news-concept, 36427887 PMC9761416

[18] Piyajaroenkij T Tantiworawit A Khikhuntod J Piriyakhuntorn P Rattanathammethee T Hantrakool S . Alteration of monocyte subsets and their functions in thalassemia patients. Int J Hematol. (2023) 117:188–97. doi: 10.1007/s12185-022-03484-9, 36323999 PMC9889407

[19] Hinjoy S Hantrakun V Kongyu S Kaewrakmuk J Wangrangsimakul T Jitsuronk S . Melioidosis in Thailand: present and future. Trop Med Infect Dis. (2018) 3:38. doi: 10.3390/tropicalmed3020038, 29725623 PMC5928800

[20] Nonejuie P Wilantho A McDonald D Htoo HH Chalerm J Tripathi A . Differential gut microbiota composition in β-thalassemia patients and its correlation with iron overload. Sci Rep. (2024) 14:23858. doi: 10.1038/s41598-024-75456-4, 39394230 PMC11470119

[21] Chung BH Ha SY Chan GC Chiang A Lee TL Ho HK . Klebsiella infection in patients with thalassemia. Clin Infect Dis. (2003) 36:575–9. doi: 10.1086/367656, 12594637

[22] Borgna-Pignatti C Rugolotto S De Stefano P Zhao H Cappellini MD Del Vecchio GC . Survival and complications in patients with thalassemia major treated with transfusion and deferoxamine. Haematologica. (2004) 89:1187–93. 15477202

[23] Jongprasartsuk W. Deep neck infections: a study of 127 cases in Nan Hospital. Lampang Med J. (2022) 32:42–50.

[24] Wangthanakorn S. Factor related to severe deep neck infections: retrospective study in Udonthani. Thai J Otolaryngol Head Neck Surg. (2021) 22:3–19.

[25] Madhok S. Dental considerations in Thalassemic patients. J Dent Med Sci. (2014) 13:57–62.

[26] Abuga KM Muriuki JM Williams TN Atkinson SH. How severe anaemia might influence the risk of invasive bacterial infections in African children. Int J Mol Sci. (2020) 21:6976. doi: 10.3390/ijms21186976, 32972031 PMC7555399

[27] Orf K Cunnington AJ. Infection-related hemolysis and susceptibility to gram-negative bacterial co-infection. Front Microbiol. (2015) 6:666. doi: 10.3389/fmicb.2015.00666, 26175727 PMC4485309

[28] Singer DB. Postsplenectomy sepsis. Perspect Pediatr Pathol. (1973) 1:285–311. 4596312

[29] Sakran W Levin C Kenes Y Colodner R Koren A. Clinical spectrum of serious bacterial infections among splenectomized patients with hemoglobinopathies in Israel: a 37-year follow-up study. Infection. (2012) 40:35–9. doi: 10.1007/s15010-011-0178-5, 21866338

